# Joint Analyses of Longitudinal and Time-to-Event Data in Research on Aging: Implications for Predicting Health and Survival

**DOI:** 10.3389/fpubh.2014.00228

**Published:** 2014-11-06

**Authors:** Konstantin G. Arbeev, Igor Akushevich, Alexander M. Kulminski, Svetlana V. Ukraintseva, Anatoliy I. Yashin

**Affiliations:** ^1^Center for Population Health and Aging, Duke University, Durham, NC, USA

**Keywords:** forecasting, mortality, health, joint model, stochastic process model, aging, trajectory

## Abstract

Longitudinal data on aging, health, and longevity provide a wealth of information to investigate different aspects of the processes of aging and development of diseases leading to death. Statistical methods aimed at analyses of time-to-event data jointly with longitudinal measurements became known as the “joint models” (JM). An important point to consider in analyses of such data in the context of studies on aging, health, and longevity is how to incorporate knowledge and theories about mechanisms and regularities of aging-related changes that accumulate in the research field into respective analytic approaches. In the absence of specific observations of longitudinal dynamics of relevant biomarkers manifesting such mechanisms and regularities, traditional approaches have a rather limited utility to estimate respective parameters that can be meaningfully interpreted from the biological point of view. A conceptual analytic framework for these purposes, the stochastic process model of aging (SPM), has been recently developed in the biodemographic literature. It incorporates available knowledge about mechanisms of aging-related changes, which may be hidden in the individual longitudinal trajectories of physiological variables and this allows for analyzing their indirect impact on risks of diseases and death. Despite, essentially, serving similar purposes, JM and SPM developed in parallel in different disciplines with very limited cross-referencing. Although there were several publications separately reviewing these two approaches, there were no publications presenting both these approaches in some detail. Here, we overview both approaches jointly and provide some new modifications of SPM. We discuss the use of stochastic processes to capture biological variation and heterogeneity in longitudinal patterns and important and promising (but still largely underused) applications of JM and SPM to predictions of individual and population mortality and health-related outcomes.

## Introduction

Longitudinal data on aging, health, and longevity provide a wealth of information to investigate different aspects of the processes of aging and development of diseases leading to death. There is a growing interest to analyses of such data not only in epidemiology but also in demographic studies. This interest is based on an increasing number of available large-scale studies that collect various biomarkers, which can be incorporated into demographic analyses (1–3). The rapid pace of advances in genetics provides additional opportunities and challenges for demographic and biodemographic applications and the need to integrate the principles of genetics and genomics into such analyses is recognized (4, 5). The ongoing efforts to incorporate genetic information into longitudinal studies is considered potentially “the most revolutionary element of the addition of biological data in large-scale surveys” (6) having far-reaching impact on the related fields of epidemiology and demography investigating interactions of genetic, biological, social, economic, and demographic characteristics (3).

Longitudinal observations of various biomarkers or physiological variables measured at different ages in the same individual allow for investigating the relationship of the dynamics of these variables and mortality or morbidity risks and getting insights about possible mechanisms and dynamics of aging-related processes. There is evidence in the literature that average age trajectories of physiological variables in short-lived individuals markedly deviate from those in long-lived individuals [see, e.g., Ref. (7, 8)]. Similar effect was also observed for average trajectories among individuals with long and short healthy lifespan (8) as well as among long- and short-lived carriers of different genetic variants, e.g., carriers/non-carriers of the APOE e4 allele (9). It was also observed that the dynamic characteristics of trajectories of biomarkers at middle and old ages (such as the rate of change, variability, the rate of decline after reaching the maximum) influence mortality risk differentiating the survival chances at older ages (8, 10, 11). These studies illustrate the importance of including longitudinal dynamics in predictive models for mortality and incidence of aging-related diseases as they provide additional information on the outcome of interest compared to baseline measurements.

Joint analysis of such longitudinal measurements and time-to-event outcomes requires a special consideration of analytical approaches. Biomarkers are collected in longitudinal studies intermittently at examination times, which may be sparse and typically biomarkers are not observed at the event times. Also, many biomarkers are subject to random biological variability. As known in the statistical literature, ignoring measurement errors and biological variation in such variables and using their “raw” values as time-dependent covariates in the Cox model may lead to biased estimates (12, 13). Statistical methods aimed at analyses of time-to-event data jointly with such longitudinal measurements became known as the “joint models (JM) for longitudinal and time-to-event data” or simply the “JM.” This is a very active area in biostatistics with a broad range of methodological developments and possible applications, see recent reviews (14–17) and a book (18).

An important point to consider in analyses of longitudinal data on aging, health, and longevity is how to incorporate knowledge and theories about mechanisms and regularities of aging-related changes that accumulate in the research field into respective analytic approaches. In the absence of specific observations of longitudinal dynamics of relevant biomarkers manifesting such mechanisms and regularities (which is a typical situation in a contemporary longitudinal studies), traditional approaches have a rather limited utility to estimate respective parameters that can be meaningfully interpreted from the biological point of view. A conceptual analytic framework that incorporates available knowledge about mechanisms of aging-related changes, which may be hidden in the individual longitudinal trajectories of physiological variables and that allows for analyzing their indirect impact on the risks of diseases and death has been recently developed in the biodemographic literature. This approach, the stochastic process model of aging (SPM), has its roots in the random-walk model by Woodbury and Manton (19). The original version of this model has been extended in various ways and applied in different contexts. These include incorporation of the notions of physiological norm, allostatic adaptation, measures of stress resistance, and adaptive capacity (20), as well as applications to analyses of age trajectories of different physiological variables (such as blood glucose, body mass index, cholesterol, diastolic blood pressure, hematocrit, pulse pressure, and pulse rate) in relation to mortality/morbidity risks (7, 8, 21, 22); applications to “indices of cumulative deficits” (23, 24); analyses of trajectories of medical costs in relation to mortality risks (25); and applications to evaluate genetic component in aging-related processes using data on candidate genes (9) and selected single-nucleotide polymorphism (SNP) alleles evaluated in genome-wide association studies (GWAS) of longevity (26). Further extensions of the basic model include the version for dependent competing risks (27); the model with hidden heterogeneity (latent classes) in longitudinal data (28); and the genetic stochastic process model for joint analyses of genotyped and non-genotyped participants of longitudinal studies (29). Yashin et al. (30) presented a comprehensive model of human aging, health, and mortality for joint analyses of data on individual health histories, age trajectories of physiological or biological variables, and mortality. This version has both jumping and continuous components that jointly have the Markov property. The jumping component represents fast changes in health status, and the continuous component describes slower individual physiological aging. The important practical value of this approach is that it provides the possibility to jointly analyze data with different structures within the same methodological framework (31, 32). Some recent developments in stochastic process models are summarized in a review paper (33).

Despite, essentially, serving similar purposes (i.e., joint analyses of longitudinal age trajectories of biomarkers and time-to-event data), these two methodologies developed in parallel in different disciplines (biostatistics and (bio)demography) with very limited cross-referencing (34, 35). Although there were several publications separately reviewing these two approaches (see previous paragraphs), there were no publications presenting both these approaches in some detail. Here, we overview both these approaches jointly as well as provide some new modifications of the SPM. We emphasize two particular aspects of these models. First, we discuss the use of stochastic processes to capture biological variation and heterogeneity in longitudinal patterns in JM. Second, we discuss important and promising (but still largely underused) applications of these approaches to predictions of individual and population mortality and health-related outcomes.

## Approaches to Joint Analyses of Longitudinal and Time-to-Event Data

### Joint models

The JM typically consist of two sub-models representing the dynamics of longitudinal (the “longitudinal sub-model”) and time-to-event, e.g., survival, data (the “survival sub-model”). It is postulated in this class of models that the observed longitudinal data are values of a “true” (unobserved) process collected intermittently and subject to measurement errors. For example, the standard model for continuous longitudinal data can be formulated using a linear mixed-effects model with normally distributed errors and random effects (18, 36–38):

(1) $$Y_{i}\left(t\right)=X_{i}^{T}\left(t\right)\beta +Z_{i}^{T}\left(t\right)b_{i}+\varepsilon_{i}\left(t\right)$$

where *Y*_i_(*t*) denotes the longitudinal outcome for individual *i* at age (or time) *t*, $X_{i}^{T}\left(t\right)$ and $Z_{i}^{T}\left(t\right)$ are design vectors of fixed effects β and random effects *b*_i_, correspondingly (“*T*” denotes transposition; here and below we will use column vectors if not stated otherwise), and ε_i_(*t*) represents the measurement error term (error terms are assumed independent and normally distributed, $\varepsilon_{i}\left(t\right)∼\;N\left(0,\sigma^{2}\right)$). Random effects *b*_i_ are assumed to be independent of ε_i_(*t*) and also normally distributed, b_i_ ~ *N*(0, *B*).

The survival sub-model specifies the expression for hazard of an event as a function of the “true” (unobserved) longitudinal outcome, e.g., using the Cox proportional hazards model:

(2) $$\mu_{i}\left(t|{\bar{Y}}_{i}\left(t\right),w_{i}\right)=\mu_{0}\left(t\right)\exp\left\{w_{i}^{T}\gamma +\alpha {\bar{Y}}_{i}\left(t\right)\right\},$$

where μ_0_(*t*) is the baseline hazard, *w*_i_ is a vector of baseline covariates and γ and α are the respective regression coefficients. Note that in Eq. 2, the hazard depends not on the observed value of a biomarker *Y*_i_(*t*) (as in the usual Cox model) but on the “true” (unobserved) value of the longitudinal outcome ${\bar{Y}}_{i}\left(t\right)$:

(3) $${\bar{Y}}_{i}\left(t\right)=X_{i}^{T}\left(t\right)\beta +Z_{i}^{T}\left(t\right)b_{i}$$

Various generalizations of such basic joint model (1–3) were suggested in the biostatistical literature during the last decades. These developments involve differences in characteristics of longitudinal and time-to-event sub-models such as specification of trajectories, distribution of random effects, type of longitudinal data (continuous or categorical), alternative expressions for hazard rates, etc. [see, e.g., recent reviews in Ref. (14–16, 18)]. Below, we will discuss in more detail particular modifications, which are especially relevant in applications to research on aging to capture effects of dynamic characteristics of individual trajectories on time-to-event outcome, to take into account biological variation and heterogeneity in longitudinal patterns of physiological variables as well as to perform predictions of individual and population mortality and health-related outcomes.

The standard JM assumes that the risks of events and longitudinal trajectories follow similar patterns for all individuals in a population (for example, a biomarker changes linearly with age for all individuals). In general, however, a population may consist of subpopulations with distinct patterns of longitudinal trajectories of biomarkers, which can also have different effects on the time-to-event outcome in each subpopulation. Also, in general, the observed covariates do not entirely capture heterogeneity in the outcomes and the population may consist of “latent subpopulations” defined by some unobserved characteristics. A special class of models, the joint latent class models, accounts for such hidden heterogeneity in the population, see review in Proust-Lima et al. (17). The joint latent class models have three components. First, the latent class indicator represents the probability of belonging to the latent class (subpopulation) specified using a multinomial logistic regression to accommodate observed covariates. The other two components are the usual longitudinal and survival sub-models but it is assumed here that individuals from different latent classes can have different patterns of longitudinal trajectories of biomarkers and different risks of event (i.e., the expressions in Eqs 1 and 2 are class-specific). The joint latent class models are more computationally feasible than the standard JM (1–3) because they do not require numerical integration in the likelihood function. These models can be efficiently used as dynamic prognostic tools that can be updated according to the observed values of the longitudinal outcome as we will discuss later in Section “Software Implementation, Limitations, and Applications to Epidemiological Studies.”

The specification of the survival sub-model (2) assumes that the hazard rate depends on the current “true” value of the longitudinal outcome. This is a reasonable assumption; however, it is evident from the literature that dynamic characteristics of the longitudinal trajectory can also affect in the risk of death or onset of a disease at a specific age. For example, such dynamic characteristics of individual trajectories as slopes, variability, or the rate of decline after reaching the maximum are related to mortality risk and risk of onset of major aging-related diseases (8, 11) and they can be better predictors of the time-to-event outcomes than the current level of the biomarker. These observations signify the importance of extensions of the JM that would allow for analyzing the dependence of the risk of an event on such dynamic characteristics of the longitudinal trajectory. Several such models have been suggested recently (39–43). Rizopoulos and Ghosh (43) presented a model with very flexible parameterization that, in particular, includes derivatives of the longitudinal profile functions. Such specification implies that the risk for an event can depend not only on the current “true” value of the longitudinal outcome but also on the dynamic characteristics (such as the slope and the curvature) of the longitudinal trajectory. Importantly, this specification, along with other generalizations, has been implemented in the R package JM (44) and extensive discussion and examples of applications are provided in the book by Rizopoulos (18). This facilitates practical applications of this approach in different research areas.

An important point to consider in applications of models is how to integrate available biological knowledge about the underlying processes and mechanisms into statistical methods. One aspect of this concerning JM is related to specifications of the longitudinal sub-model. The simpler specifications (e.g., the linear mixed-effects model) provide a convenient approximation from a computational point of view and they can be relevant in various settings. However, they may have a rather limited utility in terms of the ability to estimate parameters that can be meaningfully interpreted from the biological point of view. For example, they ignore the biological variability of individual trajectories over time and such simplification may be biologically implausible in specific applications. One possibility to capture biological variation and heterogeneity in individual longitudinal trajectories is to use stochastic processes in the longitudinal sub-model. In this case Eq 1 includes an additional term representing a stochastic process modeling the correlation between individual measurements (note that this process is distinct from measurement errors ε_i_(*t*) given by the i.i.d. random variables):

(4) $$Y_{i}\left(t\right)=X_{i}^{T}\left(t\right)\beta +Z_{i}^{T}\left(t\right)b_{i}+W_{i}\left(t\right)+\varepsilon_{i}\left(t\right)$$

where *W*_i_(*t*) is a mean zero stochastic process independent of ε_i_(*t*) and *b*_i_.

The specific choice of the process *W*_i_(*t*) can differ in applications. One particular type of stochastic processes, the Ornstein–Uhlenbeck (OU) process and its generalizations, appears to be especially relevant in epidemiological and medical applications studying mortality and morbidity risks in relation to age trajectories of biomarkers. This is because it has some appealing properties allowing for clear biological interpretation. Specifically, it has a so-called “mean reverting property,” which means that, in a long run, the OU process tends to drift toward its long-term mean. That is, the current value of the process specifies the direction of the drift: if it is less than the long-term mean value then the drift is positive (i.e., toward the mean) but if the current value is greater than this mean then the drift is negative (i.e., again, toward the mean). Such behavior of the process represents homeostatic regulation which is a fundamental property of living organisms that tend to maintain stability and return to an “equilibrium state” in case of various external and internal disturbances. Therefore, this makes the OU process (and its generalizations) a logical choice for modeling longitudinal age trajectories of biomarkers. Although there are some applications of the generalized OU process in biostatistical literature on JM (45), major developments of an approach that incorporates the underlying biological knowledge into the framework of statistical modeling appeared in the biodemographic and gerontological literature. We will present such methodology, which became known as the stochastic process models (or, alternatively, as the quadratic hazard models) in the next section.

### Stochastic process models

#### Basic model

Longitudinal studies that collect measurements of biomarkers in the same individual at different ages along with health and/or survival status are especially valuable for analyzing dynamics of physiological state, which may be associated with the process of aging leading to onset of aging-related diseases and death. However, such analyses require a conceptual analytic framework that would incorporate available knowledge about mechanisms of aging-related changes hidden in individual trajectories of biomarkers to analyze their indirect impact on mortality and morbidity risks. Although some JM are based on sound biological theories relevant to specific applications (e.g., in cancer or HIV studies), they typically lack specific parameters or components that could be interpreted in the context of aging-related mechanisms. As discussed above, one possibility to bring biological background to the statistical models is to use appropriate stochastic processes, such as the (generalized) OU process, to represent underlying biological mechanisms. Such specification allows estimating parameters that can be meaningfully interpreted from the biological point of view and thus such models are more appropriate for understanding respective biological mechanisms indirectly affecting the outcomes of interest.

The stochastic process models (20, 33) represent the age dynamics of a vector of longitudinal outcomes (e.g., physiological variables) *Y_t_* (*t* is age) by a diffusion type stochastic differential equation:

(5) $$dY_{t}=a\left(t,X\right)\left(Y_{t}-f_{1}\left(t,X\right)\right)dt+B\left(t,X\right)dW_{t}$$

with initial condition *Y*_0_. Here, *W_t_* is a vector Wiener process with independent components that models exogenous effects on the outcomes *Y_t_*. This process *W_t_* is assumed to be independent of the initial vector *Y*_0_ and a vector of (time-independent) covariates *X*. The strength of effects of *W_t_* is characterized by a matrix of diffusion coefficients *B*(*t, X*). The vector-function *f*_1_(*t, X*) represents the trajectory that the outcome *Y_t_* tends to follow (see discussion on the mean reverting property of the OU process above). Such behavior of *Y_t_* is forced by the negative feedback mechanism represented by the matrix *a*(*t, X*). This is an essential property of Eq. 5 because in the absence of such negative feedback mechanism the trajectories of *Y_t_* would deviate from *f*_1_(*t, X*) indefinitely, which is not plausible from the biological point of view if we deal with modeling living organisms subject to homeostatic regulation. The presence of *f*_1_(*t, X*) and *a*(*t, X*) in the description of the longitudinal dynamics of *Y_t_* allows for estimating two important aging-related mechanisms: the effect of allostatic adaptation [see, e.g., discussion on the concept of allostasis in Ref. (46)] and decline in the adaptive capacity [see, e.g., Ref. (47)]. Taking into account the effect of allostatic adaptation is essential when information on external disturbances or stresses affecting organisms during the life course is very limited if available at all (which is a typical situation in modern longitudinal data). Usually, the effect of decline in the adaptive capacity [i.e., in the rate of the adaptive response for deviations of *Y_t_* from the average “allostatic” trajectories *f*_1_(*t, X*)] cannot be measured directly in longitudinal studies due to the lack of appropriate biomarkers in the data. Thus, such indirect approach to analysis of this important feature of aging in the model is an important advantage for the studies on aging. More details on implementation of the concepts of allostasis and decline in adaptive capacity in the stochastic process models can be found in Ref. (20, 33).

The second equation of the model specifies the survival sub-model in the form of the risk of death (or onset of a disease) at age *t* given the value of the longitudinal outcome at that age (*Y_t_*) and a vector of time-independent observed covariates *X*. This function is assumed to be a quadratic function of the longitudinal outcome which is based on the evidence from different studies that observed U- or J-shapes of the risks as functions of various physiological variables [see, e.g., Ref. (48–55)]:

(6) $$\begin{array}{llll} \mu \;\left(t,Y_{t},X\right) & =\mu_{0}\left(t,X\right)\; & & \;\\ & \;+{\left(Y_{t}-f_{0}\left(t,X\right)\right)}^{T}Q\left(t,X\right)\left(Y_{t}-f_{0}\left(t,X\right)\right). \end{array}$$

Here μ_0_(*t, Y*_t_) is the baseline hazard, i.e., the mortality (or incidence) rate that would remain if the outcome *Y_t_* followed the optimal trajectory *f*_0_(*t, X*) and *Q*(*t, X*) is a non-negative-definite symmetric matrix of respective dimension.

The function *f*_0_(*t, X*) minimizes the risk at respective age and hence it can be interpreted as the (age-dependent) “physiological norm.” Note that, in general, this norm can be different from the “allostatic trajectories” *f*_1_(*t, X*) because the organisms function under unfavorable conditions and many such conditions affect set-points of physiological homeostasis changing physiological balance from the “normal” to “abnormal” state (56, 57). The difference between these two functions provides the measure of the allostatic load (58, 59) so the approach permits evaluating these characteristics from longitudinal data.

The component *Q*(*t, X*) can also be interpreted in terms of aging-related processes. It is related to stress resistance associated with deviations of the outcome *Y_t_* from respective norms *f*_0_(*t, X*) [see more details explaining how stress resistance is implemented in the model in Ref. (20, 22)]. Modeling stress resistance is essential in applications to the studies on aging because the literature provides compelling evidence on connections among stress resistance, aging, and longevity (60–62). It is important also that the model assumes that this component can change with age thus allowing for indirect evaluation of decline in stress resistance with age. Such indirect approach to estimate this important manifestation of aging is especially valuable when data on age dynamics of relevant biomarkers related to stress resistance are not available in longitudinal studies.

Summing up, the stochastic process model consists of the longitudinal sub-model represented by the stochastic differential equation specifying the dynamics of a vector of longitudinal outcomes and the survival (time-to-event) sub-model representing mortality (or incidence) rate as a quadratic function of the longitudinal outcomes. This model provides a conceptual analytic framework that incorporates several concepts and mechanisms relevant to research on aging such as homeostatic regulation, age-specific physiological norms, allostasis and allostatic load, decline in adaptive capacity, and stress resistance. Respective characteristics can be indirectly estimated from available information on individual age trajectories of physiological variables and follow-up information on mortality or incidence of diseases collected in longitudinal studies on aging. The basic model (5–6) can be extended in different directions, e.g., the model including hidden heterogeneity (i.e., latent subpopulations) in longitudinal data (28), the model for analyses of genetic data (29), the model for dependent competing risks (27), the model for joint analyses of individual health histories, physiological state and survival (30), and the models for joint analyses of data collected using different observational plans (31). Below, we present two modifications of the basic model, the latent class SPM and genetic SPM, with the prototype versions formulated in Yashin et al. (28) and Arbeev et al. (29).

#### Latent class SPM

The original version of the SPM that includes hidden heterogeneity (latent classes) was suggested in Yashin et al. (28). Here, we present its modification that models the latent class membership in line with recent developments in the latent class JM [see recent review in Ref. (17)]. The latent class SPM assumes that a population consists of a finite number of latent classes (subpopulations). Individuals in these homogeneous latent classes are characterized by similar patterns of longitudinal dynamics of biomarkers and their relation to the survival outcome. In different classes, however, the longitudinal patterns as well as their effect on the risk outcome can differ. Such dependence of components of the model on the latent class essentially means that all respective aging-related mechanisms may work differently in these latent subpopulations. Ignoring such hidden population structure and application of the original model can result in incorrect conclusions as the resulting effects can remain masked in the total population (28). The latent class SPM is thus a valuable tool to perform sensitivity analyses revealing the presence of such hidden population structure in the data.

Let the random variable *Z* denotes the latent class membership, that is, *Z* = *k* if an individual belongs to the class *k* = 1, …, *K*. The probabilities of the latent class membership, *p*_k_, conditional on a vector of covariates *X^0^* observed at baseline, can be specified using, e.g., a multinomial logistic regression [as in the literature on the latent class JM, see in Ref. (17, 63–65)]:

(7) $$p_{k}=P\left(Z=k|X^{0}\right)=\frac{e^{\beta_{\mathrm{0k}}+\beta_{\mathrm{1k}}^{T}X^{0}}}{1+\sum_{c=1}^{K-1}e^{\beta_{\mathrm{0c}}+\beta_{\mathrm{1c}}^{T}X^{0}}},$$

for *k* = 1, …, *K* − 1, and

(8) $$p_{\text{K}}=P\left(Z=K|X^{0}\right)=\frac{1}{1+\sum_{c=1}^{K-1}e^{\beta_{\mathrm{0c}}+\beta_{\mathrm{1c}}^{T}X^{0}}}.$$

In each latent class *k*, the age dynamics of a vector of biomarkers *Y*_t_ is given by a stochastic differential equation similar to Eq. 5 but it is assumed that all components are latent class-specific:

(9) $$dY_{t}=a^{k}\left(t,X\right)\left(Y_{t}-f_{1}^{k}\left(t,X\right)\right)dt+B^{k}\left(t,X\right)dW_{t},$$

with initial condition $Y_{t_{0}}$. Here, *X* is the vector of covariates observed at baseline that can contain some elements from *X*^0^.

The expression for the hazard rate is also similar to Eq. 6 but again with latent class-specific components:

(10) $$\begin{array}{llll} \mu^{k}\left(t,Y_{t},X\right) & =\mu_{0}^{k}\left(t,X\right)\; & & \;\\ & \;+{\left(Y_{t}-f_{0}^{k}\left(t,X\right)\right)}^{T}Q^{k}\left(t,X\right)\left(Y_{t}-f_{0}^{k}\left(t,X\right)\right). \end{array}$$

The likelihood function of the latent class SPM (8–10) is a straightforward generalization of that in the model by Yashin et al. (28) and we omit the likelihood estimation procedure here for conciseness.

#### Genetic SPM

The “genetic SPM” suggested in Arbeev et al. (29) is the version of the stochastic process model aimed at applications to longitudinal studies in which a sub-sample of participants was genotyped. This version performs joint analyses of data on genotyped and non-genotyped participants of a longitudinal study that improves power compared to analyses of non-genotyped participants alone. The model is also applicable to any other (discrete) partially observed covariate. The original version (29) can also be modified to include the dependence of the model’s components on the vector of observed (time-independent) covariates available at baseline as outlined below.

Let *k, k* = 1, …, *K*, denotes the presence of allele or genotype *k* in the genome of an individual. Similarly to expressions (7–8) for the latent class SPM, we can specify the probabilities of having this allele or genotype, *p*_k_, conditional on some vector of time-independent covariates *X*^0^ observed at baseline. The paradigm of this approach is that the presence of a specific allele or genotype can affect the dynamics of a vector of biomarkers *Y*_t_ as well as the hazard rate. For example, one can specify the expressions for age trajectories of biomarkers and hazard rate for carriers of the allele or genotype *k* as in Eqs (9–10).

Note that, although this specification looks identical to the latent class SPM, the construction of the likelihood function is different because in the latent class SPM the latent classes are not known for any individual whereas genetic data (i.e., the values *k*) are available for genotyped participants. The likelihood function for this modification of the genetic SPM is a straightforward generalization of the likelihood function for the original model in Arbeev et al. (29) and is omitted here. The likelihood function contains the same parameters in the parts for the genotyped and non-genotyped sub-samples. Thus, the addition of available information for the non-genotyped participants (i.e., the longitudinal measurements of biomarkers and time-to-event data) provides an opportunity for improving power compared to analyses based on the genotyped individuals alone. The advantage of the genetic SPM in applications to research on aging is that it has different components representing specific biological concepts and aging-related mechanisms for which the respective parameters have clear biological interpretations. This allows for testing different hypotheses on the presence of genetic effect of the alleles/genotypes on respective aging-related characteristics (such as stress resistance, adaptive capacity, age-dependent physiological norms, etc.), which is not possible in the traditional analyses.

### Software implementation, limitations, and applications to epidemiological studies

Both JM and SPM are computationally intensive and require special software implementing the parameter estimation procedures. Different research groups contributed to development of various specifications of JM. Respectively, there has been a variety of different implementations of specific variants of the models in software packages of the authors’ preference. This includes implementations in SAS [see, e.g., Ref. (39, 66–70)], Mplus (71), WinBUGS [see, e.g., Ref. (13, 42, 43, 66, 72, 73)], aML (74), and R [e.g., Ref. (44)]. Some authors provided software codes for the estimation algorithms in the publications. A recent book (18) is a useful source of practical information on the R packages (*JM* and *lcmm*) designed to fit a broad class of JM.

The general (continuous-time) SPM involves solution of the systems of ordinary differential equations (ODE) at each step of the likelihood optimization procedure. Respective computations can be performed using modern statistical and technical software such as MATLAB’s Optimization Toolbox and ODE solvers, or SAS/OR PROC OPTMODEL, implementing different optimization algorithms and methods for the ODE solution. The likelihood optimization algorithms for both continuous-time and discrete-time versions of SPM are currently implemented using MATLAB and SAS. These routines (available by request from the first author of this paper) perform parameter estimation for different parametric specifications of the models as well as allow for testing various relevant hypotheses using the likelihood ratio test for nested models and the Akaike information criterion for non-nested models. The software codes are available for the original SPM, all its modifications published in the literature, and also for the versions presented in this paper. An R package implementing the original SPM and its modifications is currently being developed.

Computational complexity of the methods (both JM and SPM) may be a limitation in certain applications. For example, GWAS data collected in longitudinal studies may contain millions of SNPs for thousands of participants. For such data, computational burden of the methods may prohibit their routine application to each SNP in the dataset, especially in high-dimensional cases. Currently, a more feasible approach in such applications is to work with pre-selected sets of SNPs (26).

Several specific computational challenges should be taken into account in practical implementations of the latent class and genetic SPMs introduced in this paper. The latent class SPM and the joint latent class models have many things in common so these challenges are similar to those discussed in the literature on the joint latent class models (17, 75). We highlight here two such challenges. First, it is known from the mixture models literature that the likelihood functions may have local maxima, which is the case for the latent class models. The recommendation is to run the estimation algorithm starting from different initial values to safeguard against convergence to a local maximum. Second, the number of latent classes is generally not known in advance in the latent class models. Therefore, it is necessary to perform sensitivity analyses with different numbers of latent classes. Also, both the latent class SPM and the genetic SPM are parametric models. Therefore, as any parametric model, they rely on the description of its components as specific parametric functions. Although the basic components of the model are all based on the solid biological theories that justify their presence in the model, their specific parametric forms are unknown and generally they cannot be empirically evaluated from the real data to guess their parametric form. Therefore, it is advisable to perform sensitivity analysis with different parametric specifications of the components of the models. Also, the specific type of genetic influence on the hidden components of aging is not known *a priori*. Thus, versions of the genetic SPM with different types of genetic influence (such as dominant, recessive, or additive form of action of the minor allele on respective characteristics) should be tested in applications. Summing up this paragraph, we note that the advanced models reviewed in this paper should never be used as a “black box” taking some data as input and producing some *p*-values as output. The failure to take into account the technical details mentioned above can result in the misinterpretation of findings and erroneous conclusions.

Another important consideration is data requirements for application of such models. These methods deal with joint analyses of longitudinal measurements and time-to-event outcomes. Therefore, they are not applicable to epidemiological studies that do not collect longitudinal measurements. It is also advisable to consider the number and frequency of available longitudinal observations before trying to apply the methods to epidemiological studies collecting both follow-up information on events and longitudinal measurements. There are no universal requirements on how often the biomarkers should be measured for the models to be applicable and the models can handle temporarily sparse data or studies with biomarkers collected in a few time points. If the dynamics of the biomarker is predictive of the time-to-event outcome then inclusion of even a limited number of measurements can potentially be advantageous compared to analyses of the baseline measurements, especially for a longer follow-up periods. However, some practical considerations need to be taken into account when specifying the models. Some biomarkers have a complex non-linear age dynamics (8, 76). For such biomarkers, more observations can be necessary to estimate that complex dynamics from the data than for those with simpler, e.g., linear, age patterns. Also, biomarkers may have different rate of change at different ages. For example, a biomarker can be relatively stable at middle ages so that even a couple of sparse observations at those ages can be representative of the dynamics in the model whereas at the old ages, when the aging-related deterioration steadily progresses (and also a substantial attrition due to mortality happens), a more frequent collection of measurements could be highly beneficial for such dynamic analyses.

In general, models for joint analyses of longitudinal and time-to-event outcomes provide more efficient estimates of the effect of a covariate (such as treatment) on time-to-event outcome in case when there is also an effect of the covariate on the trajectory of a biomarker (77). Thus, such models may require smaller sample sizes to achieve the power comparable to analyses based on time-to-event data alone. There is also an additional possibility to increase the power when some covariate (e.g., genetic marker) is available for a sub-sample of participants of a longitudinal study. For example, the genetic SPM (29) combines data from genotyped and non-genotyped participants of the longitudinal study thus increasing the power compared to analyses of genotyped participants alone. That said, we need to note that sample size and power calculations in these models is a largely underdeveloped area. For JM, Chen et al. (77) derived a closed-form sample size formula for estimating the effect of the longitudinal process, and extended Schoenfeld’s sample size formula to the joint-modeling setting for estimating the overall treatment effect. They investigated the impact of the within-subject variability on the power and data collection strategies, such as spacing and frequency of measurements, and found that different data collection strategies can substantially influence the power when the within-subject variability is large. The authors also found that optimal frequency of measurements depends on the complexity of the trajectory of biomarkers so that higher polynomial trajectories and larger measurement errors require more frequent measurements. Arbeev et al. (29) performed power calculations for the genetic SPM and showed how power increased in joint analyses of genotyped and non-genotyped participants compared to analyses of genotyped individuals alone in different scenarios. These two papers provide important contribution to this area. However, additional studies are necessary to investigate the power and sample size requirements in different settings relevant to various applications.

### Joint models as a tool for individual predictions of longitudinal and time-to-event outcomes

Available evidence in epidemiological literature suggests that the dynamic characteristics of trajectories of biomarkers observed at middle and old ages differentiate the survival chances at older ages [see, e.g., Ref. (8, 10, 11)]. It is also recognized that the longitudinal trajectories of biomarkers provide important additional information on risk of death and development of diseases compared to the baseline or current values of biomarkers. Thus, the entire trajectories of biomarkers can be used to make better predictions of the respective time-to-event and longitudinal outcomes. JM provide a framework for performing individual predictions of such outcomes. Importantly, such predictions are “dynamic” in the sense that, when additional information becomes available for the individual, the predictions can be updated taking into account this new observation. Development and application of such dynamic predictive tools is an active research area in the JM literature in recent years (13, 40, 65, 78–86). The methods predict the probability of an event in some future time period as well as the expected future value of the longitudinal outcome for an individual with available baseline information as well as observations of the longitudinal outcome. The latent class JM are more computationally attractive for these purposes than the standard JM because the former ones involve summing over latent classes whereas the latter integrate over the random effects distribution [see recent review in Ref. (17)].

Such individualized predictive tools based on JM are valuable in clinical settings for patient monitoring and decision making because such predictions can be dynamically updated according to the observations of biomarkers (83, 85, 86). Availability of free software (18, 44) and a web-based calculator (86) implementing such algorithms should facilitate the growing use of this methodology in practical applications. Such predictive tools that utilize all available information (not only that available at baseline) provide an opportunity for making more informed predictions about individual’s health; however, expectations about a “perfect individualized health forecasting” might still be unrealistic in the foreseeable future (87).

### Stochastic process models: Applications for forecasting health and mortality

Modeling and forecasting mortality in human populations is performed in different related disciplines such as demography, actuarial science, epidemiology, and statistics. Respective approaches reflect different focus of these disciplines related to specific research questions being studied. Recent reviews of available methods for projecting mortality can be found elsewhere (88–90). Demographic methods for mortality projections evolved commonly within the time-series framework working mostly with age- and year-specific death rates, e.g., the widely applied Lee-Carter model and its extensions (91). Such models usually do not include any information on risk factors found in epidemiological studies. Availability of large-scale studies collecting longitudinal data on relevant aging-related biomarkers highlights an important aspect concerning their use in such models. Specifically, one needs to take into account age dynamics of risk factors to make more relevant projections. For example, many physiological variables have complex non-linear changes with age (8, 11) so that failure to include such dynamics in the model (e.g., using only baseline values) can lead to incorrect inferences about the effects of the risk factors on mortality, and, hence, less accurate predictions and less reliable statements on how specific risk factor interventions affect mortality. This may be especially important for applications to evaluating and predicting mortality risks at advanced ages when physiological variables change rapidly with age and aging-related processes (such as decline in stress resistance and adaptive capacity) affecting risks of death develop at a quick pace.

Another aspect in mortality projections is that mortality risk is related to individual’s health status. Thus, morbidity projections are highly relevant for making projections of mortality but also they provide important insights *per se*. Several approaches for health projections have been developed and applied in different settings. For example, one of the earlier models, the Coronary Heart Disease Policy Model (92), was designed to simulate how policy changes and technological developments influence the incidence, prevalence, and mortality from coronary heart disease as well as associated changes in medical expenditures. Such microsimulation models where the modeled unit is an individual appear to be particularly convenient for evaluating the impact of different scenarios on the outcomes of interest, incorporation of data from different sources, and inclusion of assumptions about distributions of relevant variables. Dynamic microsimulation models construct individual life histories allowing behavioral and exposure changes over time. See Kopec et al. (93) and Rutter et al. (94) for recent discussion on methodological and applied issues of microsimulation approaches to simulation of diseases and health outcomes. A broader discussion on health forecasting can be found in Ref. (95, 96).

One example of dynamic continuous-time microsimulation model is the Canada’s Population Health Model (POHEM) (97, 98). The model integrates diverse information on different aspects of population health such as information on risk factors, disease onset and progression, their effects on health and functional status and so on. The model creates a “synthetic” longitudinal data set that represents the entire life of a cohort from birth to death and produces all relevant statistics from this cohort. The approach has been developed and applied to assess how different policy interventions and technological advances affect population health in Canada. In the US, the Future Elderly Model (FEM) (99, 100) is the most comprehensive microsimulation model that projects individuals’ health conditions, functional status, and health care expenditures based on a nationally representative sample of elderly individuals from the Medicare Current Beneficiary Survey (MCBS). Several “what-if” scenarios based on recommendations of a panel of experts have been considered to explore changes in the parameters of the models and the projected outcomes corresponding to different potential breakthrough technologies and changes in lifestyle and the health care system. A similar conceptual framework for using simulation methods combining information from different sources is used in the California Health Forecasting Model (101). The approach provides a full life-course model extending the age range to birth. Also, it incorporates the dynamics of population demographics (e.g., migration), and time-varying health risk factors such as physical activity and obesity that allow for better capturing the impact of public health programs and policies on the health-related outcomes of interest. The three modules of the model, the core population module, the risk factor/disease module, and the forecasting module, implement different elements of the general method, i.e., estimates of demographic and socioeconomic variables, population dynamics, estimates of exposures, risk factors, their impact on health-related outcomes, and forecasting future outcomes with or without interventions.

Forecasting approaches can vary in terms of the scope, the underlying assumptions, the modeled variables, and relationships between them and in other aspects. They can apply concepts and findings from several disciplines such as demography, epidemiology, statistics, and medicine. Comprehensive approaches may include hundreds or even thousands of parameters, for instance, age- and sex-specific mortality, incidence and birth rates, prevalence of various relevant risk factors, effects of demographic variables on the probability of exposure to these risk factors, effects of risk factors on the outcomes of interest with possible interactions, and many more. Therefore, the availability of data allowing one to estimate the parameters needed in the model as well as the use of an adequate theoretical basis is of utmost importance for constructing a relevant and credible model. The dynamic forecasting models implementing the longitudinal component require, ideally, the knowledge on how the dynamics of time-varying risk factors affects mortality and incidence rates and respective estimates should be obtained using appropriate methodology. In particular, the methods for joint analyses of longitudinal and time-to-event data reviewed here is an appropriate choice if one wishes to incorporate the dynamics of relevant biomarkers in the forecasting model.

Stochastic process models reviewed in this paper originate from the random-walk model by Woodbury and Manton (19). Theoretical developments and applications of different modifications of this model to forecasting the size, health status, and mortality of the US population have been performed since the 1980s by Manton and colleagues (102–110). Yashin et al. (103) considered how the uncertainty due to exogenous processes combined with stochasticity intrinsic to physiological aging processes propagates through time in the stochastic process model. As noted by the authors, for the purpose of forecasting, it is no longer possible to work in the framework of the conditional model because of the uncertainty of the future values of the exogenous factors and one needs to make some additional assumptions concerning the stochastic evolution of these exogenous factors. The paper considers an augmented Gaussian stochastic process model with exogenous factors influencing mortality through direct effects on the hazard function and/or through indirect effects via their influence on the evolution of endogenous physiological factors. The uncertainty of forecasts of future mortality rates is assessed by the derived expressions for the variances of the individual mortality rate and the individual survival probability. The corresponding expressions for the variability of the forecasted cohort parameters, which are due solely to the uncertainty in the evolution of the exogenous factors are obtained from these expressions by integrating out the endogenous physiological variables. Although formulated for mortality, the model also applies to other time-to-event data such as the onset of diseases. See further discussion on this approach in Stallard (109). Manton et al. (104) applied the multidimensional stochastic process model by Woodbury and Manton to forecast the size, health status, and survival of the US population. Akushevich et al. (107) developed a microsimulation strategy for the stochastic process model to forecast the effects of changes in risk factors on individual physiological changes and the risk of death. This microsimulation approach was applied to model the intervention effects of stem cell therapy on adult atherosclerosis (108) and to project future changes in the structure and health of the U.S. population due to effects of smoking on multiple outcome variables such as disease prevalence and mortality as well as fertility (110). The microsimulation approach as in Akushevich et al. (110) aggregates changes in individual event histories to produce population changes in the outcomes of interest. An important feature of such forecasting model is the ability to implement different “what-if” scenarios (similarly to, e.g., FEM).

Population health forecasting is a comprehensive task that requires rich data as well as understanding of different determinants of health and their interactions to provide an adequate output. An important aspect is that the growing availability of relevant data (e.g., longitudinal observations of physiological variables) goes in parallel with development of innovative methodology of analyzing such data. In particular, recent advances in the stochastic process model methodology allow for constructing “biologically based” forecasting models that incorporate knowledge and theories about mechanisms and regularities of aging-related changes accumulated in the literature. Such models contain parameters and components, which can be meaningfully interpreted in biological terms relevant to research on aging. Thus, they allow simulating the effects of interventions aimed at reducing or postponing aging-related changes on mortality or incidence of diseases. The approach has a capability to accommodate several additional sources of information. Genetic data can be implemented either as separate genetic variants or aggregated “genetic risk scores” that have been shown to significantly influence mortality risk and age trajectories of various physiological variables (26, 111, 112). These data can be analyzed either in the frameworks of the Yashin et al. (20) model or in the genetic stochastic process model (29). Indices of cumulative deficits (23, 113) provide an efficient approach to investigate aging-related processes of health deterioration. Such indices are shown to better characterize susceptibility to death in elderly people than phenotypic frailty (114) and they appear to be a more important determinant of long-term risks of death and longevity than physiological variables (115). The cumulative indices can be implemented in the stochastic process model as in our earlier work (24). An alternative approach to accommodate the impact of systemic dysregulation in an organism on mortality risk is to use the recently developed measure of physiological dysregulation (116) in the stochastic process model (respective modification of the model will be reported elsewhere).

A recently developed comprehensive approach for joint analyses of data on individual health histories, age trajectories of physiological variables, and mortality has been presented in Yashin et al. (30). This version of the stochastic process model has both jumping and continuous components representing fast changes in health status and slower individual physiological aging respectively. The important practical value of the developed approach is that it provides the possibility for joint analysis of data with different structures (e.g., discrete-time observations of continuously changing physiological variables with unobserved changes in health status, or unmeasured physiological variables but observed health transitions, or a combination of the above) within the same methodological framework (31, 32). Such approach combines data from different sources that has an obvious advantage because separate “incomplete” datasets cannot provide estimates of all parameters needed, for example, for forecasting models. This comprehensive model is a unique tool to connect trajectories of physiological variables with data on health status and mortality that has parameters interpretable in terms relevant to studies on aging. The most detailed specification of the model can incorporate diverse information about genetic and non-genetic factors including pleiotropic, polygenic, and age-specific effects of genes on health and survival, as well as dynamic mechanisms of aging-related changes evaluated from longitudinally measured physiological variables [see review of recent advances in these research topics relevant for use in forecasting models in (117)].

## Conclusion

We reviewed recent methodologies relevant for performing joint analyses of longitudinal and time-to-event data: the JM and the stochastic process models. These active research areas have a broad range of recent methodological developments and possible applications. We presented two new modifications of the stochastic process models, the latent class and genetic stochastic process models that generalize previous prototype versions developed in earlier publications. Among many possible applications of the JM and the stochastic process models, we focused on applications to predictions of individual and population mortality and health-related outcomes. JM provide a flexible framework for performing individual predictions of such outcomes which is also valuable in clinical settings for patient monitoring and decision making. Availability of free software and web-based tools implementing respective algorithms is valuable for practical applications. Recent advances in the stochastic process model methodology allow for constructing “biologically based” forecasting models that incorporate knowledge and theories about mechanisms and regularities of aging-related changes accumulated in the literature. Such models provide a tool to connect trajectories of physiological variables and data on health status and mortality with parameters interpretable in terms relevant to studies on aging. The approach also allows for combining data from different sources with different structures, which is especially valuable when no single data set contains all required information to fit such a comprehensive model.

## Conflict of Interest Statement

The authors declare that the research was conducted in the absence of any commercial or financial relationships that could be construed as a potential conflict of interest.

## References

[1] Weinstein M Vaupel JW Wachter KW Biosocial Surveys. Washington, DC: The National Academies Press (2007). 21977539

[2] Crimmins E Vasunilashorn S Kim JK Alley D Biomarkers related to aging in human populations. Adv Clin Chem (2008) 46:161–216 10.1016/S0065-2423(08)00405-8 19004190 PMC5938178

[3] Crimmins E Kim JK Vasunilashorn S. Biodemography: new approaches to understanding trends and differences in population health and mortality. Demography (2010) 47:S41–64. 10.1353/dem.2010.0005 21302421 PMC5870619

[4] Carey JR Biodemography: research prospects and directions. Demogr Res (2008) 19:1749–57 10.4054/DemRes.2008.19.50

[5] Wachter KW. Biodemography comes of age. Demogr Res (2008) 19:1501–12. 10.4054/DemRes.2008.19.40 19633726 PMC2714645

[6] Suzman R Prologue: research on the demography and economics of aging. Demography (2010) 47:S1–4 10.1353/dem.2010.0013 21302427 PMC5870621

[7] Yashin AI Ukraintseva SV Arbeev KG Akushevich I Arbeeva LS Kulminski AM. Maintaining physiological state for exceptional survival: what is the normal level of blood glucose and does it change with age? Mech Ageing Dev (2009) 130:611–8. 10.1016/j.mad.2009.07.004 19635493 PMC2764411

[8] Yashin AI Arbeev KG Ukraintseva SV Akushevich I Kulminski A Patterns of aging related changes on the way to 100: an approach to studying aging, mortality, and longevity from longitudinal data. North Am Actuar J (2012) 16:403–33 10.1080/10920277.2012.10597640 PMC5070546 27773987

[9] Arbeev KG Ukraintseva SV Kulminski AM Akushevich I Arbeeva LS Culminskaya IV Effect of the APOE polymorphism and age trajectories of physiological variables on mortality: application of genetic stochastic process model of aging. Scientifica (2012) 2012:568628. 10.6064/2012/568628 23682334 PMC3653307

[10] Van Vliet P Oleksik AM Van Heemst D De Craen AJM Westendorp RGJ. Dynamics of traditional metabolic risk factors associate with specific causes of death in old age. J Gerontol A Biol Sci Med Sci (2010) 65:488–94. 10.1093/gerona/glq014 20154178

[11] Yashin AI Arbeev KG Akushevich I Arbeeva L Kravchenko J Il’yasova D Dynamic determinants of longevity and exceptional health. Curr Gerontol Geriatr Res (2010) 2010:381637. 10.1155/2010/381637 20953403 PMC2952789

[12] Prentice RL Covariate measurement errors and parameter estimation in a failure time regression model. Biometrika (1982) 69:331–42 10.1093/biomet/69.2.331

[13] Sweeting MJ Thompson SG. Joint modelling of longitudinal and time-to-event data with application to predicting abdominal aortic aneurysm growth and rupture. Biom J (2011) 53:750–63. 10.1002/bimj.201100052 21834127 PMC3443386

[14] Sousa I A review on joint modelling of longitudinal measurements and time-to-event. Revstat Stat J (2011) 9:57–81.

[15] Wu L Liu W Yi GY Huang Y Analysis of longitudinal and survival data: joint modeling, inference methods, and issues. J Probab Stat (2012) 2012:640153 10.1155/2012/640153

[16] Mccrink LM Marshall AH Cairns KJ Advances in joint modelling: a review of recent developments with application to the survival of end stage renal disease patients. Int Stat Rev (2013) 81:249–69 10.1111/insr.12018

[17] Proust-Lima C Sene M Taylor JM Jacqmin-Gadda H. Joint latent class models for longitudinal and time-to-event data: a review. Stat Methods Med Res (2014) 23:74–90. 10.1177/0962280212445839 22517270 PMC5863083

[18] Rizopoulos D Joint Models for Longitudinal and Time-to-Event Data With Applications in R. Boca Raton, FL: Chapman and Hall/CRC (2012).

[19] Woodbury MA Manton KG A random-walk model of human mortality and aging. Theor Popul Biol (1977) 11:37–48 10.1016/0040-5809(77)90005-3 854860

[20] Yashin AI Arbeev KG Akushevich I Kulminski A Akushevich L Ukraintseva SV. Stochastic model for analysis of longitudinal data on aging and mortality. Math Biosci (2007) 208:538–51. 10.1016/j.mbs.2006.11.006 17300818 PMC2084381

[21] Yashin AI Arbeev KG Akushevich I Ukraintseva SV Kulminski A Arbeeva LS Exceptional survivors have lower age trajectories of blood glucose: lessons from longitudinal data. Biogerontology (2010) 11:257–65. 10.1007/s10522-009-9243-1 19644762 PMC3351103

[22] Arbeev KG Ukraintseva SV Akushevich I Kulminski AM Arbeeva LS Akushevich L Age trajectories of physiological indices in relation to healthy life course. Mech Ageing Dev (2011) 132:93–102. 10.1016/j.mad.2011.01.001 21262255 PMC3064744

[23] Kulminski A Yashin A Arbeev K Akushevich I Ukraintseva S Land K Cumulative index of health disorders as an indicator of aging-associated processes in the elderly: results from analyses of the national long term care survey. Mech Ageing Dev (2007) 128:250–8. 10.1016/j.mad.2006.12.004 17223183 PMC1866299

[24] Yashin AI Arbeev KG Kulminski A Akushevich I Akushevich L Ukraintseva SV. Health decline, aging and mortality: how are they related? Biogerontology (2007) 8:291–302. 10.1007/s10522-006-9073-3 17242962 PMC1995090

[25] Yashin AI Arbeev KG Kulminski A Akushevich I Akushevich L Ukraintseva SV. What age trajectories of cumulative deficits and medical costs tell us about individual aging and mortality risk: findings from the NLTCS-Medicare data. Mech Ageing Dev (2008) 129:191–200. 10.1016/j.mad.2007.12.005 18242665 PMC2289773

[26] Yashin AI Arbeev KG Wu D Arbeeva LS Kulminski A Akushevich I How lifespan associated genes modulate aging changes: lessons from analysis of longitudinal data. Front Genet (2013) 4:3. 10.3389/fgene.2013.00003 23346098 PMC3551204

[27] Akushevich I Arbeev K Ukraintseva S Yashin A Theory of individual health histories and dependent competing risks. JSM Proc Sect Risk Anal (2011):5385–99.

[28] Yashin AI Arbeev KG Akushevich I Kulminski A Akushevich L Ukraintseva SV Model of hidden heterogeneity in longitudinal data. Theor Popul Biol (2008) 73:1–10 10.1016/j.tpb.2007.09.001 17977568 PMC2268646

[29] Arbeev KG Akushevich I Kulminski AM Arbeeva LS Akushevich L Ukraintseva SV Genetic model for longitudinal studies of aging, health, and longevity and its potential application to incomplete data. J Theor Biol (2009) 258:103–11. 10.1016/j.jtbi.2009.01.023 19490866 PMC2691861

[30] Yashin AI Akushevich I Arbeev KG Kulminski A Ukraintseva S. Joint analysis of health histories, physiological states, and survival. Math Popul Stud (2011) 18:207–33. 10.1016/S0025-6196(11)60487-419720775

[31] Yashin AI Akushevich I Arbeev KG Kulminski A Ukraintseva SV New approach for analyzing longitudinal data on health, physiological state, and survival collected using different observational plans. JSM Proc Sect Gov Stat (2011):5336–50.

[32] Yashin AI Akushevich I Arbeev K Kulminski A Ukraintseva S Chapter 19. Methodological aspects of studying human aging, health, and mortality. In: Hoque N Mcgehee MA Bradshaw BS, editors. Applied Demography and Public Health. Dordrecht: Springer (2013). p. 337–55.

[33] Yashin AI Arbeev KG Akushevich I Kulminski A Ukraintseva SV Stallard E The quadratic hazard model for analyzing longitudinal data on aging, health, and the life span. Phys Life Rev (2012) 9:177–88 10.1016/j.plrev.2012.05.002 22633776 PMC3392540

[34] Martinussen T Keiding N. The manton-woodbury model for longitudinal data with dropouts. Stat Med (1997) 16:273–83. 10.1002/(SICI)1097-0258(19970215)16:3<273::AID-SIM485>3.0.CO;2-4 9004397

[35] Aalen OO Borgan O Gjessing HK Survival and Event History Analysis: A Process Point of View. New York, USA: Springer (2008).

[36] Faucett CL Thomas DC. Simultaneously modelling censored survival data and repeatedly measured covariates: a Gibbs sampling approach. Stat Med (1996) 15:1663–85. 10.1002/(SICI)1097-0258(19960815)15:15<1663::AID-SIM294>3.0.CO;2-1 8858789

[37] Wulfsohn MS Tsiatis AA. A joint model for survival and longitudinal data measured with error. Biometrics (1997) 53:330–9. 10.2307/2533118 9147598

[38] Tsiatis AA Davidian M Joint modeling of longitudinal and time-to-event data: an overview. Stat Sin (2004) 14:809–34.

[39] Ye W Lin X Taylor JMG. Semiparametric modeling of longitudinal measurements and time-to-event data – a two-stage regression calibration approach. Biometrics (2008) 64:1238–46. 10.1111/j.1541-0420.2007.00983.x 18261160

[40] Yu M Taylor JMG Sandler HM Individual prediction in prostate cancer studies using a joint longitudinal survival-cure model. J Am Stat Assoc (2008) 103:178–87 10.1198/016214507000000400

[41] Brown ER. Assessing the association between trends in a biomarker and risk of event with an application in pediatric HIV/AIDS. Ann Appl Stat (2009) 3:1163–82. 10.1214/09-AOAS251 20802852 PMC2928653

[42] Gao F Miller JP Xiong C Beiser JA Gordon M The Ocular Hypertension Treatment Study. A joint-modeling approach to assess the impact of biomarker variability on the risk of developing clinical outcome. Stat Methods Appt (2011) 20:83–100. 10.1007/s10260-010-0150-z 21339862 PMC3039885

[43] Rizopoulos D Ghosh P. A Bayesian semiparametric multivariate joint model for multiple longitudinal outcomes and a time-to-event. Stat Med (2011) 30:1366–80. 10.1002/sim.4205 21337596

[44] Rizopoulos D JM: an R package for the joint modelling of longitudinal and time-to-event data. J Stat Softw (2010) 35:1–33.21603108

[45] Struthers CA Mcleish DL. A particular diffusion model for incomplete longitudinal data: application to the multicenter AIDS cohort study. Biostatistics (2011) 12:493–505. 10.1093/biostatistics/kxq079 21199891

[46] Mcewen BS Wingfield JC. The concept of allostasis in biology and biomedicine. Horm Behav (2003) 43:2–15. 10.1016/S0018-506X(02)00024-7 12614627

[47] Hall DM Xu L Drake VJ Oberley LW Oberley TD Moseley PL Aging reduces adaptive capacity and stress protein expression in the liver after heat stress. J Appl Physiol (2000) 89:749–59. 10926662 10.1152/jappl.2000.89.2.749

[48] Allison DB Faith MS Heo M Kotler DP. Hypothesis concerning the U-shaped relation between body mass index and mortality. Am J Epidemiol (1997) 146:339–49. 10.1093/oxfordjournals.aje.a009275 9270413

[49] Okumiya K Matsubayashi K Wada T Fujisawa M Osaki Y Doi Y A U-shaped association between home systolic blood pressure and four-year mortality in community-dwelling older men. J Am Geriatr Soc (1999) 47:1415–21. 10591234 10.1111/j.1532-5415.1999.tb01559.x

[50] Boutitie F Gueyffier F Pocock S Fagard R Boissel JP. J-shaped relationship between blood pressure and mortality in hypertensive patients: new insights from a meta-analysis of individual-patient data. Ann Intern Med (2002) 136:438–48. 10.7326/0003-4819-136-6-200203190-00007 11900496

[51] Mazza A Zamboni S Rizzato E Pessina AC Tikhonoff V Schiavon L Serum uric acid shows a J-shaped trend with coronary mortality in non-insulin-dependent diabetic elderly people. The cardiovascular study in the elderly (CASTEL). Acta Diabetol (2007) 44:99–105. 10.1007/s00592-007-0249-3 17721747

[52] Protogerou AD Safar ME Iaria P Safar H Le Dudal K Filipovsky J Diastolic blood pressure and mortality in the elderly with cardiovascular disease. Hypertension (2007) 50:172–80. 10.1161/HYPERTENSIONAHA.107.089797 17515449

[53] Kulminski AM Arbeev KG Kulminskaya IV Ukraintseva SV Land K Akushevich I Body mass index and nine-year mortality in disabled and nondisabled older U.S. individuals. J Am Geriatr Soc (2008) 56:105–10. 10.1111/j.1532-5415.2007.01494.x 18005352

[54] Kuzuya M Enoki H Iwata M Hasegawa J Hirakawa Y J-shaped relationship between resting pulse rate and all-cause mortality in community-dwelling older people with disabilities. J Am Geriatr Soc (2008) 56:367–8 10.1111/j.1532-5415.2007.01512.x 18251825

[55] Van Uffelen JGZ Berecki-Gisolf J Brown WJ Dobson AJ. What is a healthy body mass index for women in their seventies? Results from the Australian longitudinal study on women’s health. J Gerontol A Biol Sci Med Sci (2010) 65:844–50. 10.1093/gerona/glq058 20413529

[56] Sterling P Eyer J Allostasis: a new paradigm to explain arousal pathology. In: Fisher S Reason J, editors. Handbook of Life Stress, Cognition and Health. New York: John Wiley & Sons (1988). p. 629–49.

[57] Mcewen BS Allostasis and allostatic load: implications for neuropsychopharmacology. Neuropsychopharmacology (2000) 22:108–24 10.1016/S0893-133X(99)00129-3 10649824

[58] Seeman TE Mcewen BS Rowe JW Singer BH. Allostatic load as a marker of cumulative biological risk: MacArthur studies of successful aging. Proc Natl Acad Sci U S A (2001) 98:4770–5. 10.1073/pnas.081072698 11287659 PMC31909

[59] Karlamangla AS Singer BH Seeman TE. Reduction in allostatic load in older adults is associated with lower all-cause mortality risk: MacArthur studies of successful aging. Psychosom Med (2006) 68:500–7. 10.1097/01.psy.0000221270.93985.82 16738085

[60] Parsons PA. The ecological stress theory of aging and hormesis: an energetic evolutionary model. Biogerontology (2007) 8:233–42. 10.1007/s10522-007-9080-z 17473992

[61] Vermeulen CJ Loeschcke V. Longevity and the stress response in *Drosophila*. Exp Gerontol (2007) 42:153–9. 10.1016/j.exger.2006.09.014 17110070

[62] Le Bourg E Hormesis, aging and longevity. Biochim Biophys Acta (2009) 1790:1030–9 10.1016/j.bbagen.2009.01.004 19463497

[63] Lin HQ Turnbull BW Mcculloch CE Slate EH Latent class models for joint analysis of longitudinal biomarker and event process data: application to longitudinal prostate-specific antigen readings and prostate cancer. J Am Stat Assoc (2002) 97:53–65 10.1198/016214502753479220

[64] Proust-Lima C Joly P Dartigues J-F Jacqmin-Gadda H Joint modelling of multivariate longitudinal outcomes and a time-to-event: a nonlinear latent class approach. Comput Stat Data Anal (2009) 53:1142–54 10.1016/j.csda.2008.10.017

[65] Proust-Lima C Taylor JMG. Development and validation of a dynamic prognostic tool for prostate cancer recurrence using repeated measures of posttreatment PSA: a joint modeling approach. Biostatistics (2009) 10:535–49. 10.1093/biostatistics/kxp009 19369642 PMC2697347

[66] Guo X Carlin BP Separate and joint modeling of longitudinal and event time data using standard computer packages. Am Stat (2004) 58:16–24 10.1198/0003130042854

[67] Vonesh EF Greene T Schluchter MD. Shared parameter models for the joint analysis of longitudinal data and event times. Stat Med (2006) 25:143–63. 10.1002/sim.2249 16025541

[68] Liu L Huang X O’quigley J. Analysis of longitudinal data in the presence of informative observational times and a dependent terminal event, with application to medical cost data. Biometrics (2008) 64:950–8. 10.1111/j.1541-0420.2007.00954.x 18162110

[69] Liu L. Joint modeling longitudinal semi-continuous data and survival, with application to longitudinal medical cost data. Stat Med (2009) 28:972–86. 10.1002/sim.3497 19040210

[70] Gueorguieva R Rosenheck R Lin H. Joint modelling of longitudinal outcome and interval-censored competing risk dropout in a schizophrenia clinical trial. J R Stat Soc Ser A Stat Soc (2012) 175:417–33. 10.1111/j.1467-985X.2011.00719.x 22468033 PMC3315284

[71] Wang P Shen W Boye ME. Joint modeling of longitudinal outcomes and survival using latent growth modeling approach in a mesothelioma trial. Health Serv Outcomes Res Methodol (2012) 12:182–99. 10.1007/s10742-012-0092-z 22773919 PMC3384782

[72] Hatfield LA Boye ME Carlin BP. Joint modeling of multiple longitudinal patient-reported outcomes and survival. J Biopharm Stat (2011) 21:971–91. 10.1080/10543406.2011.590922 21830926 PMC3212950

[73] Huang Y Dagne G Wu L. Bayesian inference on joint models of HIV dynamics for time-to-event and longitudinal data with skewness and covariate measurement errors. Stat Med (2011) 30:2930–46. 10.1002/sim.4321 21805486

[74] Liu L Ma JZ O’quigley J. Joint analysis of multi-level repeated measures data and survival: an application to the end stage renal disease (ESRD) data. Stat Med (2008) 27:5679–91. 10.1002/sim.3392 18693300

[75] Han J Starting values for EM estimation of latent class joint model. Commun Stat Simul Comput (2009) 38:1519–34 10.1080/03610910903019913

[76] Yashin AI Akushevich IV Arbeev KG Akushevich L Ukraintseva SV Kulminski A. Insights on aging and exceptional longevity from longitudinal data: novel findings from the Framingham heart study. Age (2006) 28:363–74. 10.1007/s11357-006-9023-7 17895962 PMC1994150

[77] Chen LM Ibrahim JG Chu H. Sample size and power determination in joint modeling of longitudinal and survival data. Stat Med (2011) 30:2295–309. 10.1002/sim.4263 21590793 PMC3278672

[78] Taylor JMG Yu MG Sandler HM. Individualized predictions of disease progression following radiation therapy for prostate cancer. J Clin Oncol (2005) 23:816–25. 10.1200/JCO.2005.12.156 15681526

[79] Garre FG Zwinderman AH Geskus RB Sijpkens YWJ A joint latent class changepoint model to improve the prediction of time to graft failure. J R Stat Soc Ser A Stat Soc (2008) 171:299–308 10.1111/j.1467-985X.2007.00514.x

[80] Hanson TE Branscum AJ Johnson WO Predictive comparison of joint longitudinal-survival modeling: a case study illustrating competing approaches. Lifetime Data Anal (2011) 17:3–28 10.1007/s10985-010-9162-0 20369294

[81] Rizopoulos D. Dynamic predictions and prospective accuracy in joint models for longitudinal and time-to-event data. Biometrics (2011) 67:819–29. 10.1111/j.1541-0420.2010.01546.x 21306352

[82] Hatfield LA Carlin BP Clinically relevant graphical predictions from Bayesian joint longitudinal-survival models. Health Serv Outcomes Res Methodol (2012) 12:169–81 10.1007/s10742-012-0087-9

[83] Li H Gatsonis C Dynamic optimal strategy for monitoring disease recurrence. Sci China Math (2012) 55:1565–82 10.1007/s11425-012-4475-y PMC4269482 25530747

[84] Mauguen A Rachet B Mathoulin-Pelissier S Macgrogan G Laurent A Rondeau V. Dynamic prediction of risk of death using history of cancer recurrences in joint frailty models. Stat Med (2013) 32:5366–80. 10.1002/sim.5980 24030899

[85] Njagi EN Rizopoulos D Molenberghs G Dendale P Willekens K A joint survival-longitudinal modelling approach for the dynamic prediction of rehospitalization in telemonitored chronic heart failure patients. Stat Modelling (2013) 13:179–98 10.1177/1471082X13478880

[86] Taylor JMG Park Y Ankerst DP Proust-Lima C Williams S Kestin L Real-time individual predictions of prostate cancer recurrence using joint models. Biometrics (2013) 69:206–13. 10.1111/j.1541-0420.2012.01823.x 23379600 PMC3622120

[87] Ioannidis JPA Limits to forecasting in personalized medicine: an overview. Int J Forecast (2009) 25:773–83 10.1016/j.ijforecast.2009.05.003

[88] Booth H Demographic forecasting: 1980 to 2005 in review. Int J Forecast (2006) 22:547–81 10.1016/j.ijforecast.2006.04.001

[89] Booth H Tickle L Mortality modelling and forecasting: a review of methods. Ann Actuar Sci (2008) 3:3–43 10.1017/S1748499500000440

[90] Siegel JS Demography and Epidemiology of Human Health and Aging. New York: Springer (2012).

[91] Lee RD Carter LR Modeling and forecasting united states mortality. J Am Stat Assoc (1992) 87:659–71 10.2307/2290201

[92] Weinstein MC Coxson PG Williams LW Pass TM Stason WB Goldman L. Forecasting coronary heart disease incidence, mortality, and cost: the coronary heart disease policy model. Am J Public Health (1987) 77:1417–26. 10.2105/AJPH.77.11.1417 3661794 PMC1647098

[93] Kopec JA Fines P Manuel DG Buckeridge DL Flanagan WM Oderkirk J Validation of population-based disease simulation models: a review of concepts and methods. BMC Public Health (2010) 10:710. 10.1186/1471-2458-10-710 21087466 PMC3001435

[94] Rutter CM Zaslavsky AM Feuer EJ. Dynamic microsimulation models for health outcomes: a review. Med Decis Making (2011) 31:10–8. 10.1177/0272989X10369005 20484091 PMC3404886

[95] Soyiri IN Reidpath DD. Evolving forecasting classifications and applications in health forecasting. Int J Gen Med (2012) 5:381–9. 10.2147/IJGM.S31079 22615533 PMC3355849

[96] Soyiri IN Reidpath DD An overview of health forecasting. Environ Health Prev Med (2013) 18:1–9 10.1007/s12199-012-0294-6 22949173 PMC3541816

[97] Wolfson MC. POHEM: a framework for understanding and modelling the health of human populations. World Health Stat Q (1994) 47:157–76. 7740830

[98] Will BP Berthelot JM Nobrega KM Flanagan W Evans WK. Canada’s population health model (POHEM): a tool for performing economic evaluations of cancer control interventions. Eur J Cancer (2001) 37:1797–804. 10.1016/S0959-8049(01)00204-0 11549434

[99] Goldman DP Shekelle PG Bhattacharya J Hurd M Joyce GF Lakdawalla DN Health Status and Medical Treatment of the Future Elderly: Final Report. TR-169-CMS. Santa Monica, CA: RAND Corporation (2004).

[100] Goldman DP Shang BP Hattacharya J Garber AM Hurd M Joyce GF Consequences of health trends and medical innovation for the future elderly. Health Aff (2005) 24:W5R5–17. 10.1377/hlthaff.w5.r5 16186147 PMC6205231

[101] Van Meijgaard J Fielding JE Kominski GF. Assessing and forecasting population health: integrating knowledge and beliefs in a comprehensive framework. Public Health Rep (2009) 124:778–89. 19894419 10.1177/003335490912400604 PMC2773940

[102] Manton KG Stallard E Chronic Disease Risk Modelling: Measurement and Evaluation of the Risks of Chronic Disease Processes. London, UK: Charles Griffin Limited (1988).

[103] Yashin AI Manton KG Stallard E. The propagation of uncertainty in human mortality processes operating in stochastic environments. Theor Popul Biol (1989) 35:119–41. 10.1016/0040-5809(89)90013-0 2727949

[104] Manton KG Stallard E Singer B. Projecting the future size and health status of the united states elderly population. Int J Forecast (1992) 8:433–58. 10.1016/0169-2070(92)90057-G 12157867

[105] Manton KG Singer BH Suzman R Forecasting the Health of Elderly Populations. New York: Springer-Verlag (1993).

[106] Singer BH Manton KG. The effects of health changes on projections of health service needs for the elderly population of the United States. Proc Natl Acad Sci U S A (1998) 95:15618–22. 10.1073/pnas.95.26.15618 9861019 PMC28093

[107] Akushevich I Kulminski A Manton K Life tables with covariates: dynamic model for nonlinear analysis of longitudinal data. Math Popul Stud (2005) 12:51–80 10.1080/08898480590932296

[108] Kravchenko J Goldschmidt-Clermont PJ Powell T Stallard E Akushevich I Cuffe MS Endothelial progenitor cell therapy for atherosclerosis: the philosopher’s stone for an aging population? Sci Aging Knowledge Environ (2005) 2005:e18–18. 10.1126/sageke.2005.25.pe18 15975898

[109] Stallard E Demographic issues in longevity risk analysis. J Risk Insur (2006) 73:575–609 10.1111/j.1539-6975.2006.00190.x

[110] Akushevich I Kravchenko JS Manton KG. Health-based population forecasting: effects of smoking on mortality and fertility. Risk Anal (2007) 27:467–82. 10.1111/j.1539-6924.2007.00898.x 17511712

[111] Yashin AI Wu D Arbeev KG Stallard E Land KC Ukraintseva SV. How genes influence life span: the biodemography of human survival. Rejuvenation Res (2012) 15:374–80. 10.1089/rej.2011.1290 22607627 PMC3419845

[112] Yashin AI Wu D Arbeev KG Ukraintseva SV. Polygenic effects of common single-nucleotide polymorphisms on life span: when association meets causality. Rejuvenation Res (2012) 15:381–94. 10.1089/rej.2011.1257 22533364 PMC3419841

[113] Mitnitski AB Mogilner AJ Rockwood K. Accumulation of deficits as a proxy measure of aging. ScientificWorldJournal (2001) 1:323–36. 10.1100/tsw.2001.58 12806071 PMC6084020

[114] Kulminski AM Ukraintseva SV Kulminskaya IV Arbeev KG Land K Yashin AI. Cumulative deficits better characterize susceptibility to death in elderly people than phenotypic frailty: lessons from the cardiovascular health study. J Am Geriatr Soc (2008) 56:898–903. 10.1111/j.1532-5415.2008.01656.x 18363679 PMC2703425

[115] Kulminski AM Ukraintseva SV Culminskaya IV Arbeev KG Land KC Akushevich L Cumulative deficits and physiological indices as predictors of mortality and long life. J Gerontol A Biol Sci Med Sci (2008) 63:1053–9. 10.1093/gerona/63.10.1053 18948555 PMC2684458

[116] Cohen AA Milot E Yong J Seplaki CL Fueloep T Bandeen-Roche K A novel statistical approach shows evidence for multi-system physiological dysregulation during aging. Mech Ageing Dev (2013) 134:110–7. 10.1016/j.mad.2013.01.004 23376244 PMC3971434

[117] Arbeev KG Akushevich I Kulminski AM Ukraintseva SV Yashin AI Biodemographic analyses of longitudinal data on aging, health, and longevity: recent advances and future perspectives. Adv Geriatr (2014) 2014:957073 10.1155/2014/957073 PMC4290867 25590047

